# Fever

**Published:** 1828-07

**Authors:** 


### HOSPITAL REPORTS,

*(Principally condensed from various Periodical Publications.)*

### FEVER.

### *Cases of Fever, in which the Hydrochloruret of Lime was used, by* Dr. Reid, *at the* Fever and Dysentery Hospital, Dublin.

I.—James Doyle, admitted on the 8th of March, having been thirteen days ill of fever. He complained of severe headache; bowels costive. Having put him under a treatment consisting of cold applications of vinegar and water to his head, giving him the saline diaphoretic mixture every second or third hour, with occasional employment of purgatives when necessary, he became apparently convalescent on the 16th.

On the 22d, he complained of pain under the sternum, which I relieved by the application of eight leeches, and a little saline diaphoretic mixture.

On the 25th, he had a complete relapse of fever. By putting him under proper treatment, which consisted principally of mercurial medicines, on the 29th he became again convalescent. A slight cough, however, still remained; for which he was directed an expectorating mixture.

2d April.—A friend having been permitted to see this patient yesterday, through mistaken kindness induced him to drink some wine. In the night he complained of most violent pain in his head. He now lies nearly insensible. Low muttering delirium. Tongue deep yellow, dry, and furred; great subsultus.

Half an ounce of the mixture, with Hydrochloruret of Lime and Tincture of Columbo, was directed to be given to him every second hour.

3d.—Much better in all respects; general warm perspiration, not profuse; but still some delirium and subsultus.

Perstet.

4th.—Tongue cleaning; subsultus gone. No delirium.

5th.—Still continues to improve. Tongue dry, and he cannot put it outside his teeth, though he apparently makes considerable effort when desired to do so.

I directed a blister to his neck, and to continue the medicine as before.

6th —Much fœtid discharge from the blister. Continues to improve; can now freely put out his tongue when desired. It is now moist and clean at the edges. He has some appetite today.

7th.—Still improving.

The Diaphoretic Mixture was now substituted for that with the Hydrochloruret.

12th.—Up and dressed, sitting on his bedside. Well.

This was one of the most remarkable cases I have witnessed of the efficacy of the hydrochloruret; whether it be considered with reference to the severity of the disease, or to the rapidity of the patient's recovery.

II.—H. C. was nine days ill of fever on the 19th March. She was supposed to have taken the infection by going into lodgings where fever had been previously; but did not know this circumstance until the 18th. She appears of very delicate form, light complexion, and much marked with the small-pox. Complains of great pain in left side, headache, flushing; pulse extremely quick, rather ganglionic. Tongue dry and furred.

**I directed to have twelve leeches applied to her side, and to take an ounce of the Saline Diaphoretic Mixture three times a day.**

From this treatment some relief was obtained: however, on the 22d, cough and the febrile symptoms were increased; the headache was severe. On the 24th, a red papular eruption appeared very general over the chest, arms, and back; the intervening skin seemed white and natural; febrile symptoms still increasing; great debility; low delirium; great coldness of the extremities; bowels free, but evacuations very dark-coloured.

**Half an ounce of the mixture with the Hydrochloruret of Lime was directed to be taken three times a day.**

25th.—Tongue moist; easy sleep this morning; extremities warm. No apparent change in the eruption.

27th.—Eruption nearly gone; stools now natural. Has continued steadily getting better since 24th. Still some headache; tongue clean. The indications for employing the hydrochloruret having now disappeared, she was directed the saline diaphoretic medicine; and on the 31st she was convalescent.

She continued gathering strength until the 5th April, when a swelling of the right ear attracted notice. On the 6th, the swelling was increased, and erysipelas became developed. This was treated as usual in such cases, and on the 15th her convalescence was complete.

III.—Benjamin Hepinstall was two days ill of fever. On his admission, 13th March, he complained of pain in the abdomen, which was extremely tender on pressure, and he exhibited an expression of considerable anxiety. These symptoms being relieved, his head became much affected. On the 18th, he was apparently convalescent. On the 19th, however, the ganglionic disease again made its appearance. The former treatment was adopted, and leeches were applied to the abdomen, without apparently having any efficacy towards arresting the progress of the disease.

On the 22d, he lay on his back. There was almost total prostration of strength; pulse scarcely perceptible; occasional cough. The entire surface of the body, limbs, and tunica albuginea of the eyes, of a dirty yellow colour. A dull purple circumscribed colour in the cheeks; tongue dry and loaded. Considering this patient in a proper stage of the disease to derive benefit from the Hydrochloruret of Lime, a mixture with this medicine was directed to be taken three times a day.

23d.—After taking the medicine three times yesterday, he had a quiet night. Slept well. Tongue now cleansing and moist.

Yellowness of the skin not apparently changed, but the purple flush has disappeared from his cheeks. Pulse full and calm. He continued to mend steadily under this treatment until the 26th, when all symptoms of ganglionic disease being gone, his pulse was become spinal, and he complained of great pain in his back.—Finding that the mixture with the Hydrochloruret of Lime had apparently no effect upon these symptoms, I reverted to the usual treatment in such cases, and on the 31st March he was discharged cured.

IV.—The following case exhibits the influence of the Hydrochloruret of Lime in controlling disease of the pulmonary organs, resulting from febrile excitement.

Ann Lenon was admitted into the fever ward No. V. on the 7th February, 1827. She complained of great headache, pain in her bowels, and other febrile symptoms, in such severity as to require to be bled largely on two successive days.

On the 10th, she was getting worse, and a blister was applied over the whole abdomen.

On the 11th, better.

13th.—Abdominal pain quite relieved, but complained of severe pain now in her chest. A blister was applied with some benefit.

16th.—The pain in her chest being still severe, another blister was applied, and a pectoral mixture was ordered.

17th.—Breathing extremely difficult, which induced Dr. Brereton, whose patient she then was, to direct that she should again be bled.

By this bleeding she was much relieved, and, her improvement continuing progressive, on the 20th she was remitted to the convalescent ward.

She continued apparently to recover strength until the 6th March; she then complained of pain and sense of fulness in her chest. I directed leeches to be applied, from which she derived some relief. She complained, however, of sense of constriction about her throat. Soon afterwards she began to expectorate a considerable quantity of thick viscid mucus. Countenance bloated. As the disease advanced, in a few days she became affected with severe rigor, which sometimes continued for several hours. Her voice became nearly extinct. At length she could only swallow liquids, and was threatened with suffocation at every moment.

It would be unnecessary to detail the treatment which I adopted up to this period: it was varied accordingly as the circumstances of the case appeared to require. No plan of treatment, however, which I put in force, appeared to have any influence in arresting the progress of the disease.

On the 24th March, the state of her case was as follows: She had rigor every day since the 17th; voice and appetite almost gone; mucous rattle in breathing; could take no food, but had constant thirst, which she attempted to relieve by supping cold water; expectoration thick, and got up with difficulty; cheeks bloated with purple hue; tongue furred.

On the 25th, I directed the mixture with Hydrochloruret of Lime every second hour.

29th.—She appeared to get better on the 26th, and continued steadily to improve: her appetite was returning, her tongue was clean and moist, and since the 25th she had not a rigor. Having persevered in the same treatment a few days longer, and all the bad symptoms having apparently subsided, I thought it advisable to diminish the quantity of the mixture to one dose daily. She soon, however, changed for the worse, the bad symptoms threatened to return, and I found it necessary again to direct the medicine three times a day. This I continued till all danger of relapse seemed over; and on the 3d April her convalescence was complete.*

---

### DYSENTERY.

* From the Transactions of the Association of the Fellows and Licentiates of the King and Queen's College of Physicians in Ireland.

